# The ion channel transient receptor potential melastatin-2 does not play a role in inflammatory mouse models of chronic obstructive pulmonary diseases

**DOI:** 10.1186/1465-9921-13-30

**Published:** 2012-04-04

**Authors:** Liz Hardaker, Parmjit Bahra, Benjamin Cochin de Billy, Mark Freeman, Natalia Kupfer, Daniel Wyss, Alexandre Trifilieff

**Affiliations:** 1 Novartis Institutes for BioMedical Research, Respiratory Diseases Area, Horsham, UK; 2 Novartis Institutes for BioMedical Research, Respiratory Diseases Area, Basel, Switzerland

**Keywords:** Transient receptor potential melastatin-2, Oxidative stress, Lung inflammation

## Abstract

**Background:**

There is strong evidence that oxidative stress is associated with the pathogenesis of chronic obstructive pulmonary disease (COPD). The transient receptor potential melastatin-2 (TRPM2) is an oxidative stress sensing channel that is expressed in a number of inflammatory cells and therefore it has been suggested that inhibition of TRPM2 could lead to a beneficial effect in COPD patients. In this study, we have investigated the role of TRPM2 in a variety of mouse models of oxidative stress and COPD using TRPM2-deficent mice.

**Methods:**

Mice were exposed to ozone (3 ppm for 4 h) or lipopolysaccharide (LPS, 0.3 mg/kg, intranasaly). In another model, mice were exposed to tobacco smoke (750 μg/l total wet particulate matter) for 30 min twice a day on three consecutive days. For the exacerbation model, the smoke exposure on the morning of day 3 animals was replaced with intranasal administration of LPS (0.3 mg/kg). Animals were killed 3 and 24 h after the challenge (ozone and LPS model) or 18 h after the last tobacco smoke exposure. In vitro neutrophil chemotaxis and monocyte activation were also studied using cells isolated from wild type and TRPM2-deficient animals. Statistical significance for the in vivo data (*P *< 0.05) was determined using analysis of variance with Kruskal-Wallis and Dunns multiple comparison test.

**Results:**

In all models studied, no difference in the bronchoalveolar lavage inflammation could be evidenced when comparing wild type and TRPM2-deficient mice. In addition, no difference could be seen in the lung inflammation as assessed by the measurement of various cytokines/chemokines. Similarly in various in vitro cellular activation assays using isolated neutrophils and monocytes no significant differences could be observed when comparing wild type and TRPM2-deficient mice.

**Discussion:**

We have shown, in all the models tested, no difference in the development of airway inflammation or cell activation between TRPM2-deficient mice and their wild type counterparts. These results would suggest that inhibiting TRPM2 activity in COPD would have no anti-inflammatory effect.

## Background

Chronic obstructive pulmonary disease (COPD) is a multifactorial disease involving inflammation, mucus production dysregulation, lung parenchymal destruction and systemic effects, all of which contribute to airflow limitation. In developed countries, cigarette smoking is the main aetiological agent of COPD and is believed to account for approximately 90% of the cases [1]. The burden of COPD on the healthcare system is enormous and it is predicted that COPD will become the third leading cause of death and the fourth commonest cause of disability in the world by 2020 [2]. Despite that, pharmacotherapies available to these patients are mainly symptomatic and none of them have been shown to alter the course of the disease [3]. There is strong evidence that oxidative stress from cigarette smoking is associated with the pathogenesis of COPD. In addition to this exogenous source of oxidative stress, the chronic inflammation that takes place in the lungs of COPD patients also contributes to the oxidative stress and the pathogenesis of COPD [4]. This would suggest that targeting the oxidative stress burden in COPD patients could lead to beneficial effect.

The vertebrate transient receptor potential ion channel family is divided into six subfamilies; ankyrin (TRPAs), canonical (TRPCs), melastatin (TRPM), mucolipin (TRPMLs), polycystickidneydisease (TRPPs) and vanilloid (TRPVs). This classification is based on protein sequence homology and all family members have six putative transmembrane domains and a pore region between the fifth and the sixth transmembrane domains. These proteins assemble into homo- or hetero-tetramers to form active cation channels [5]. A number of TRP subfamilies serve as a sensor for a variety of chemical (menthol or acrolein) and physical stimuli such as oxidative stress, temperature, membrane potential changes and osmotic stress. Three of the subfamilies have members reported to play a role in the oxidative stress sensing. They are TRPA, TRPM and TRPV [6]. Among the oxidative stress sensors, TRPM2 is of interest since it has been shown to be expressed in a number of inflammatory cells (i.e., neutrophil, macrophage, monocyte). In addition, TRPM2 has also been shown to be activated by pro-inflammatory stimuli such as lipopolysaccharide (LPS, a major component of the outer cell wall of Gram-negative bacteria) and TNF-α. Since all the above inflammatory cells and stimuli are thought to be of importance in COPD pathogenesis, it has been suggested that inhibition of TRPM2 could lead to a beneficial effect in COPD patients [5].

In this study, we have investigated the role of TRPM2 in a variety of mouse models of COPD and in vitro cell-based assays using TRPM2-deficient mice [7]. Our results show, in all the models tested, no difference in the development of airway inflammation or cell activation between TRPM2-deficient mice and their wild type counterpart. These results would suggest that inhibiting TRPM2 activity in COPD would have no anti-inflammatory effect.

## Methods

### Animals

Male and female TRPM2-deficient mice [7] and their wild type counterparts were backcrossed onto BALB/C using the speed congenics service, MAX-BAX^® ^(Charles River). The genotype was confirmed using DNA isolated from blood samples. Animals were kept at an ambient temperature of 22 ± 2°C under a 12 hours normal phase light-dark cycle. Food and drinking water was freely available and animals were acclimatized for a period of at least 7 days upon arrival in the animal house prior to initiation of any experimental work. The ozone exposure experiments were carried out at Novartis Basel (Switzerland) with the approval of the Veterinary Authority of the City of Basel (Kantonales Veterinaeramt, Basel-Stadt). All other experiments were carried out at Novartis Horsham (UK) under a Project License issued by the United Kingdom Home Office under the Animal (Scientific Procedures) Act 1986 and approved by local ethical review processes.

### Ozone exposure

The protocol was modified from Williams et al. [8]. Unrestrained mice were placed in plastic exposure chamber of the following dimensions: 23 cm wide, 48 cm deep and 17 cm height (0.1 m2) (maximum of 5 mice per chamber) and exposed to 3 parts per million (ppm) ozone or room air for 4 hours. Ozone was generated, at a flow of 480 l/h, and monitored using a computer-controlled ozone simulator system (SIM6050-T; Anseros GmbH, Germany). Animals were killed 3 and 24 hours post exposure.

### LPS challenge

Mice were challenged intranasally, as previously described [9], with 0.3 mg/kg of lipopolysaccharide (*E. coli serotype 0111:B4) *or its vehicle (isotonic saline, 25 μl). Animals were killed at 3 and 24 hours post challenge.

### Tobacco smoke exposure and exacerbation models

The protocol was modified from our previous studies performed in rats [10]. Unrestrained mice were placed in plastic wholebody exposure chambers (maximum of 10 mice per chamber) and exposed to mainstream tobacco smoke for 30 minutes, twice a day on three consecutive days with at least a 5 hours gap between each exposure. Tobacco smoke was generated, at a flow rate of 0.6 l/min, using 1R3F Kentucky Research cigarettes and monitored using an EMMS (Bordon UK) total particulate matter transducer (mean wet total particulate matter of 406 ± 7 μg/l). Sham animals were exposed to room air only. For the exacerbation model, on the morning of day 3 animals were administered LPS (*E. coli serotype 0111:B4*; 0.3 mg/ml or saline, intranasally). Five hours after the LPS challenge, animals were exposed to tobacco smoke for 30 minutes. In both models, animals were killed 18 hours after the last tobacco smoke exposure.

### Assessment of airway inflammation

At the indicated time point, terminal anesthesia was induced with pentobarbitone sodium (60 mg/kg, intraperitoneally). The trachea was cannulated and bronchoalveolar lavage (BAL) was performed by instilling g 3 times 0.4 ml of phosphate buffered saline into the lung via the trachea. Total cell counts were measured and cytospins prepared. Cells were stained with Diff-Quik and a differential count of 200 cells performed using standard morphological criteria. Immediately after BAL was performed, the top right lung lobe was removed and snap frozen in liquid nitrogen for subsequent analysis. This lung lobe was homogenized in phosphate buffered saline containing a protease inhibitor cocktail (Roche, UK). For the ozone experiment, CXCL1, CXCL2, IL-6 (R&D Systems, Abingdon, UK and plasma albumin (Bethyl laboratories, Montgomery, TX, USA) were determined using ELISAs according to the manufacturer's instructions. For the tobacco smoke experiments cytokine analysis was performed using 7-plex pro-inflammatory cytokine mesoscale discovery (MSD) assay following manufacturer's instructions. Lactate dehydrogenase activity was assessed using a Cytotoxicity detection kit following manufacturer's instructions (Roche, UK).

### In vitro functional assays

Peripheral blood was collected from the abdominal vena cava of mouse. Pooled blood from 4 mice was used to assess neutrophil shape change. Agonists (10 μl) were added to 80 μl of blood for 5 minutes at 37°C followed by fixation with 2.5% paraformaldehyde (Cell Fix™, BD). Red blood cells were lysed using ice cold lysis buffer (1.5 mM NH_4_Cl and 0.1 mM KHC0_3_). Forward scatter/side scatter (FSC/SSC) plots were used to gate granulocytes (FACSCalibur, Becton Dickinson). The gated neutrophils were distinguished in FSC/FL2 plots and the mean FSC value was used as a measure of shape change.

Mouse bone marrow neutrophils were isolated from femurs and tibias from 4 mice. Bone marrow cells were pelleted by centrifugation and suspended in 5 ml of PBS. Thirteen ml of 65% Percoll (GE Healthcare) was layered on to 13 ml of a 75% Percoll solution. The cell suspension was layered on top of the gradient. After centrifugation at 1600 × *g *for 30 min, the neutrophil fraction between the 75 and 65% layer was collected. HTS-transwell-96 well plates with 3 μm pore size membranes (Costar) were used to determine chemotaxis of calcein-AM (0.5uM) labelled neutrophils. The medium containing chemoattractant was added to the lower chamber. Neutrophils (5 × 10^5 ^cells) were allowed to migrate from the upper to lower chamber for 1 hour and the number of migrating cells was quantitated by reading the lower plate on a fluorescence plate reader (BioTek Synergy) at 485 nm excitation and 520 nm emission.

Mouse bone marrow monocytes were isolated from femurs and tibias from 4 mice. Bone marrow cells were pelleted by centrifugation. The collected bone marrow cells were counted and the monocytes were isolated by CD11b positivity using a magnetic activated cell sorting system (Miltenyi Biotech). CD11b^+ ^cells were seeded into 96 well plates at 1.10^5 ^per well in RPMI 1640 medium (supplemented with 10% fetal bovine serum, 5 mM L-glutamine, 30 units/ml penicillin, and 30 mg/ml streptomycin). Cells were stimulated with LPS (*E. Coli, 055:B5*) for a period of 6 or 24 hours. Cell free supernatants were collected and assayed for CXCL2 (R&D Systems Duo set ELISA kit) and IL-10, IL-6 and TNF-α (Mesoscale Discovery).

### Data analysis

Data are expressed as mean ± standard error of the mean (SEM). Statistical significance (*P *< 0.05) was determined by analysis of variance with Kruskal-Wallis and Dunns multiple comparison test (GraphPad Software, San Diego, CA, USA).

## Results and discussion

### In vivo models

**Figure 1:**
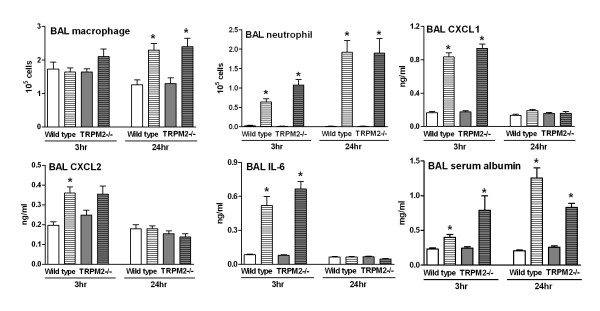
**TRPM2 deficiency has no effect on the ozone-induced BAL inflammation**. TRPM2-deficient mice (grey bars) or their wild type counterpart (white bars) were exposed to ozone (3 ppm for 4 hours, hatched bars) or air (plain bars) as described in methods section. Animals were killed for bronchoalveolar lavage differential cell counts and soluble mediator measurement at 3 and 24 hours after the end of exposure. Data are shown as mean ± SEM of 2 different experiments, each including 10-11 animals per group. Significance (*P *< 0.05), indicated by *, is against the air-exposed mice for each strain. No statistical significance was observed between deficient and wild type animals on all the parameters measured.

TRPM2 is expressed in various inflammatory cells (i.e. neutrophil, macrophage and monocyte) and has been implicated in oxidative stress-induced inflammatory response in these cell types [11]. Therefore, we compared the response of wild type and TRPM2-deficient mice in a model of ozone-induced airway inflammation. At 3 hours post ozone exposure, when compared to air-exposed animals, wild type mice had an increased number of BAL neutrophils together with elevated levels of BAL CXCL1, CXCL2, IL-6 and serum albumin (a marker of plasma leakage). At 24 hours post ozone exposure, neutrophil numbers and serum albumin levels were still elevated, whereas CXCL1, CXCL2 and IL-6 levels had returned to baseline. BAL macrophage numbers were only elevated at the 24 hours time point. At both time points and for all the parameters measured no difference could be observed when comparing the wild type mice with TRPM2-deficient mice (Figure 1).

**Figure 2:**
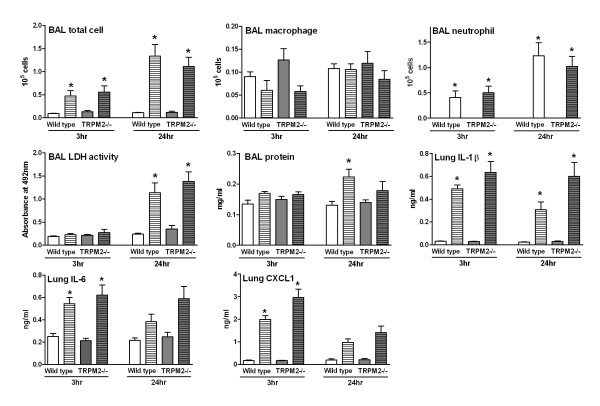
**TRPM2 deficiency has no effect on the LPS-induced BAL inflammation and lung cytokine production**. TRPM2-deficient mice (gray bars) or their wild type counterpart (white bars) were challenged with LPS (hatched bars) or saline (plain bars) as described in methods. Animals were killed for bronchoalveolar lavage differential cell counts and lung soluble mediator measurement at 3 and 24 hours post challenge. Data are shown as mean ± SEM of 7-8 animals per group. Significance (*P *< 0.05), indicated by *, is against the air-exposed mice for each strain. No statistical significance was observed between deficient and wild type animals on all the parameters measured.

**Figure 3:**
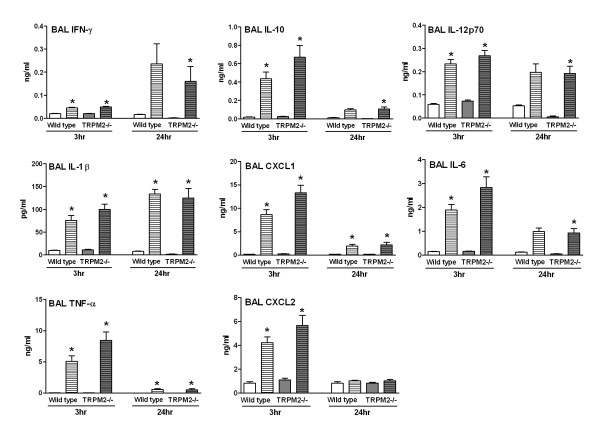
**TRPM2 deficiency has no effect on the LPS-induced BAL cytokine production**. TRPM2-deficient mice (gray bars) or their wild type counterpart (white bars) were challenged with LPS (hatched bars) or saline (plain bars) as described in methods. Animals were killed for bronchoalveolar lavage soluble mediator measurement at 3 and 24 hours post challenge. Data are shown as mean ± SEM of 7-8 animals per group. Significance (*P *< 0.05), indicated by *, is against the air-exposed mice for each strain. No statistical significance was observed between deficient and wild type animals on all the parameters measured.

Since no differences were observed between wild type and TRPM2-deficient mice in an exogenous model of oxidative stress-induced airway inflammation, we went on to study the possible role of TRPM2 in a mechanistic model of endogenously mediated oxidative stress (i.e. infiltrating inflammatory cells following LPS challenge). At 3 hours post LPS challenge, when compared to saline-challenged animals, wild type mice had an increased number of BAL total cells and neutrophil numbers together with increased levels of lung IL-1β, IL-6 and CXCL1. At 24 hours post challenge, BAL neutrophil and total cell numbers as well as lung IL-1β and IL-6 levels were still elevated. BAL lactate dehydrogenase (LDH) activity (a marker of tissue damage) and protein levels (a marker of lung edema) were only elevated at the 24 hours time point. BAL macrophage numbers were not affected by the LPS challenge (Figure 2). At 3 hours post LPS challenge the following cytokine/chemokines were also increased in the BAL: IFN-γ, IL-10, IL-12p70, IL-1β, CXCL1, IL-6, TNF-α and CXCL2. The levels of BAL IL-10, CXCL1, IL-6 and TNF-α decreased towards baseline but were still significantly increased, whereas the levels of CXCL2 had returned to baseline. Levels of IFN-γ, IL-12p70 and IL-1β were either increased or sustained at the 24 hours time point (Figure 3). At both time points and for all the parameters measured no difference could be observed when comparing the wild type mice with TRPM2-deficient mice (Figures 2 &3). During the preparation of this manuscript, a report studying the lung inflammatory response in TRPM2-deficient mice following endotoxin challenge was published. In contrast to our data, the authors reported an increased production of lung chemokines/cytokines in the lung of the TRPM2-deficient mice when compared to their wild type counterpart as well as an increased sequestration of neutrophils into the lung [12]. A major difference between this recent study [12] and our work is the route of administration for LPS. We have used intranasal dosing whereas Di and colleagues [12] used intraperitoneal dosing. This could explain the differences observed between the two studies as the LPS-induced lung inflammation is highly dependent on the route of dosing. As such, only following local lung application of LPS can neutrophils be detected in the airway space [13]. Another difference between the two studies is that we have monitored the cellular inflammation in the BAL, whereas Di and colleagues used lung myeloperoxidase as a surrogate for neutrophil influx. In addition, Di and colleagues have used C57BL/6 mice, a strain we have shown to be a lower responder to LPS when compared to BALB/c [9]. Put together, all these differences could explain the apparent discrepancy between the two studies.

**Figure 4:**
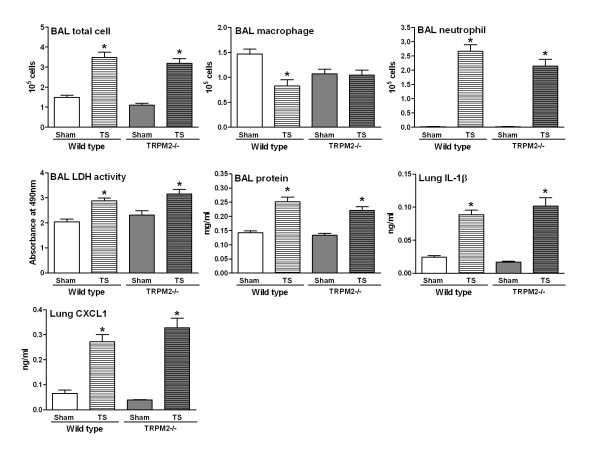
**TRPM2 deficiency has no effect on the tobacco smoke-induced BAL inflammation and lung cytokine production**. TRPM2-deficient mice (gray bars) or their wild type counterpart (white bars) were exposed to tobacco smoke (hatched bars) or air (plain bars) as described in methods. Animals were killed for bronchoalveolar lavage differential cell counts and bronchoalveolar lavage and lung soluble mediator measurement 18 hours after the end of last exposure. Data are shown as mean ± SEM of 10-11 animals per group. Significance (*P *< 0.05), indicated by *, is against the air-exposed mice for each strain. No statistical significance was observed between deficient and wild type animals on all the parameters measured.

**Figure 5:**
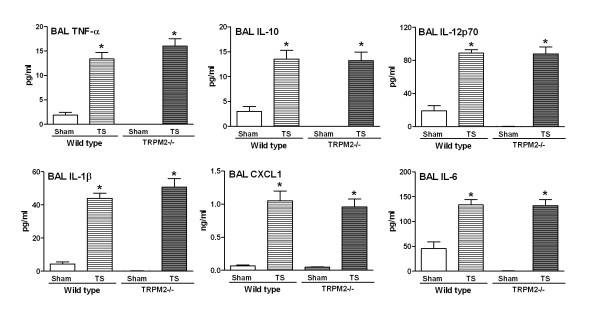
**TRPM2 deficiency has no effect on the tobacco smoke-induced BAL cytokine production**. TRPM2-deficient mice (gray bars) or their wild type counterpart (white bars) were exposed to tobacco smoke (hatched bars) or air (plain bars) as described in methods. Animals were killed for bronchoalveolar lavage soluble mediator measurement 18 hours after the end of last exposure. Data are shown as mean ± SEM of 10-11 animals per group. Significance (*P *< 0.05), indicated by *, is against the air-exposed mice for each strain. No statistical significance was observed between deficient and wild type animals on all the parameters measured.

**Figure 6:**
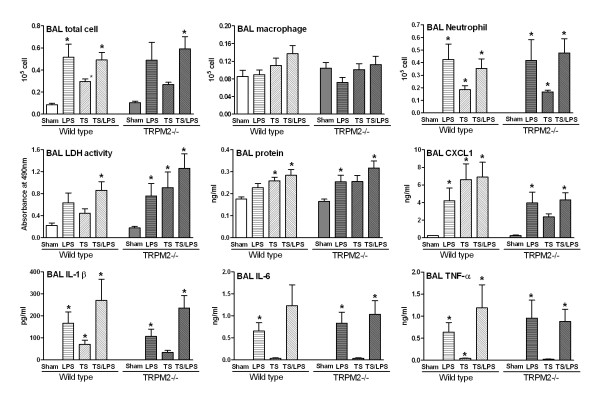
**TRPM2 deficiency has no effect on the tobacco smoke/LPS-induced BAL inflammation**. TRPM2-deficient mice (gray bars) or their wild type counterpart (white bars) were sham-exposed (plain bars) or exposed to LPS (horizontal hatched bars), tobacco smoke (left hatched bars, TS) or their combination (right hatched bars, TS/LPS) as described in methods. Animals were killed for bronchoalveolar lavage differential cell counts and soluble mediator measurement 18 hours after the end of last exposure. Data are shown as mean ± SEM of 9-11 animals per group. Significance (*P *< 0.05), indicated by *, is against the air-exposed mice for each strain. No statistical significance was observed between deficient and wild type animals on all the parameters measured.

In view of the lack of effect of TRPM2 deficiency in the above mechanistic models of oxidative stress-induced airway inflammation, we went on to compare the response of wild type and TRPM2-deficient mice in a more disease relevant model and used tobacco smoke to induce lung inflammation. When compared to air-exposed wild type mice, animals exposed to tobacco smoke had elevated BAL total cell and neutrophil numbers and a decreased number of BAL macrophages. BAL LDH activity and protein levels as well as lung levels of IL-1β and CXCL1 were elevated (Figure 4). Tobacco smoke exposure also increased the levels of BAL IL-10, IL-12p70, IL-1β, CXCL1, IL-6 and TNF-α (Figure 5). For all the parameters measured no differences were found when comparing the wild type mice with TRPM2-deficient mice (Figure 4 &5). Finally, an exacerbation model combining tobacco smoke exposure and LPS challenge was investigated. In agreement with the experiments reported above (Figures 2, 3, 4, 5), exposure to LPS or tobacco smoke alone induced a BAL inflammation that was not significantly different between wild type mice and TRPM2-deficient mice (Figure 6). When both stimuli were combined, the increase in BAL cellular inflammation as well as LDH activity, protein and CXCL1 levels were mainly driven by the LPS challenge. An additive effect was seen for the levels of BAL IL-1β, IL-6 and TNF-α. For all the parameters measured, in the mice exposed to tobacco smoke and LPS, no difference was observed when comparing the wild type mice with TRPM2-deficient mice (Figure 6).

Utilizing different models of either exogenous or endogenous oxidative stress-induced airway inflammation, we could not demonstrate a role for TRPM2 in the inflammatory response. This is somewhat in contrast to a recent publication showing that TRPM2-deficient mice had an attenuated neutrophil infiltration and CXCL2 production in a dextran sulfate sodium-induced ulcerative colitis model [7]. However, it is interesting to note that the beneficial effect observed with the TRPM2-deficient mice was almost exclusively driven by an inhibition of CXCL2 production and other pro-inflammatory cytokines such as IL-6 or several CCL chemokines were not affected [7]. This would suggest that TRPM2 inhibition would not have a general anti-inflammatory effect but rather selectively inhibits some of the pro-inflammatory mediators. The results obtained in the dextran sulfate sodium model are not surprising and in line with the published literature. Indeed, the production and release of reactive oxygen species by immune cells play a crucial role in the development of colitis [14] and CXCL2 has a detrimental role in this model [15] whereas other neutrophil chemoattractants such as CXCL1 have a protective role [16]. This is in contrast to several mouse airways inflammatory models where both CXCL1 and CXCL2 have been shown to have a crucial role in the development of the neutrophilic inflammation [17]. Based on these observations, one would expect a selective inhibition of CXCL2 in models of airway inflammatory diseases not to translate into a meaningful effect on the overall inflammation. Nevertheless, in our in vivo models, no difference in CXCL2 production or release of other pro-inflammatory mediators was observed between wild type mice and TRPM2-deficient mice. In order to further investigate these discrepancies, we studied the role of TRPM2 in in vitro assays of neutrophil chemotaxis and CD11 + cells activation.

### In vitro neutrophil chemotaxis and CD11^+ ^cells activation

**Figure 7:**
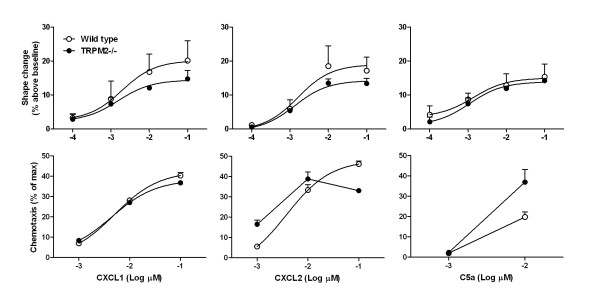
**TRPM2 deficiency has no effect on blood neutrophil shape change and chemotaxis to various stimuli**. Neutrophils were isolated from either TRPM2-deficient mice (plain circle) or their wild type counterpart (open circle) and shape change, using whole blood, or chemotaxis, using purified neutrophil from bone marrow, to various stimuli was measured. Data are shown as mean ± SEM of 3 different experiments.

We first investigated whether TRPM2 plays a role in neutrophil chemotaxis induced by various stimuli. Using whole blood from wild type mice, a concentration-dependent neutrophil shape change was observed when the cells were stimulated with CXCL1, CXCL2 and C5a. For all the stimuli used, no difference could be detected when comparing neutrophils from wild type and TRPM2-deficient mice. The same stimuli also induced chemotaxis of neutrophils, isolated from the bone marrow, and again no difference could be observed when comparing neutrophils from wild type or TRPM2-deficient mice (Figure 7). These results are in agreement with the published literature and confirm that CXCL2-induced neutrophil chemotaxis is not dependent on TRPM2 activation [17]. They also extend these results, in that CXCL1, another important airway neutrophil chemoattractant [17], does not require TRPM2 to induce neutrophil chemotaxis [7].

**Figure 8:**
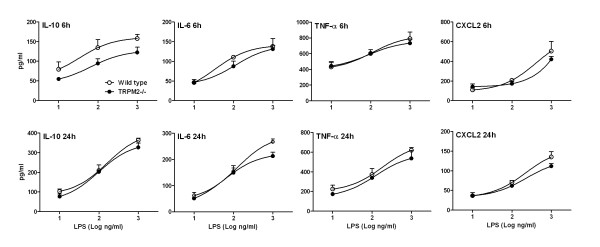
**TRPM2 deficiency has no effect on LPS-induced CD11+ cells activation**. Bone marrow CD11+ cells were isolated from either TRPM2-deficient mice (plain circle) or their wild type counterpart (open circle) and stimulated with various stimuli for 6 or 24 hours for measurement of pro-inflammatory mediators. Data are shown as mean ± SEM of 3 different experiments.

Since monocytes/macrophages are also key cells in the development of the airway inflammation in the in vivo models used above, we studied whether TRPM2 would have any involvement in their activation driven by oxidative stress. H_2_O_2_-induced a concentration-dependent (3-300 μM) Ca^2+ ^influx in CD11^+ ^cells purified from both wild type and TRPM2-deficient mice. A 31% decrease in the Ca^2+ ^signal was observed in the TRPM2-deficient monocyte, but only when stimulated with 10 μM H_2_O_2 _(data not shown). H_2_O_2 _stimulation (100 μM) also induced the release of CXCL2 that was reduced by about 40% in the deficient mice when compared to wild type animals (in pg/ml: wild type, 61.2 ± 20.4; deficient, 37.8 ± 5.2, not significant). Similarly, when using another oxidative stimuli, 10% cigarette smoke condensate, a numerical decrease in the levels of CXCL2 released from the CD11^+ ^cells isolated from deficient mice was observed (in ng/ml: wild type, 1.01 ± 0.25; deficient, 0.75 ± 0.07, not significant). These data are in agreement with previously published results describing a partial inhibition of H_2_O_2_-induced CXCL2 production from CD11^+ ^cells isolated from TRPM2-deficient mice [7]. However, using LPS as a stimulus we could not confirm a role for TRPM2 in CD11^+ ^cells activation [7]. In our hands, LPS induced a concentration- and time-dependent release of IL-10, IL-6, TNF-α and CXCL2 from mouse CD11^+ ^cells. The same stimuli also induced activation of CD11^+ ^cells isolated from TRPM2-deficient mice and no difference could be evidenced when comparing cells isolated from wild type mice with TRPM2-deficient mice (Figure 8).

Although, the in vitro experiments described above suggest some difference in the activation of CD11^+ ^cells when comparing wild type mice with TRPM2-deficient mice, these differences were minute and stimuli-dependent. It is therefore unsurprising that they do not translate into a relevant physiological effect. Hence, our in vitro data is supportive of the lack of effect of TRPM2 deficiency observed in all the in vivo models of oxidative stress-induced lung inflammation used in the present study. Since several studies have linked TRPM2 activation and oxidative stress [11], the lack of effect of TRPM2 deficiency in our in vivo and in vitro models could seems somewhat surprising. However, a closer look at the literature might well support our data. In addition to the CXCL2-selective effect described above [7], in the human monocytic cell line THP-1, only a partial inhibition of LPS-induced pro-inflammatory cytokine release was observed following downregulation of TRPM2 using shRNA despite a full inhibition of the LPS-induced intracellular calcium increase [18]. In human neutrophils, it was shown that fMLP-induced calcium influx and chemotaxis was TRPM2 dependent [19,20]. In contrast stimulation of human neutrophil with PAF was independent of TRPM2 [20]. Consistent with the human situation, fMLP- but not CXCL2-induced calcium and migration responses were reduced in neutrophils from TRPM2-deficient mice [7]. All these data, including ours, suggest that the contribution of TRPM2 to neutrophil and/or monocyte responses is highly dependent on the stimuli but also on the read out studied. In addition, more recent data suggest that another ion channel, I_CRAC_, that is expressed in neutrophils [21], is more important than TRPM2 in mediating intracellular calcium increase in responses to H_2_O_2 _[22].

## Conclusion

We have shown that in a mechanistic model of oxidative stress-induced lung injury (ozone exposure) and in several models thought to reproduce aspects of the inflammation observed in COPD, TRPM2-deficiency did not affect the development of airway inflammation. These data suggest that TRPM2 blockers may not have beneficial effects in patients with COPD.

## Abbreviations

BAL: Bronchoalveolar lavage; COPD: Chronic obstructive pulmonary disease; LDH: Lactate dehydrogenase; LPS: Lipopolysaccharide; SEM: Standard error of the mean; TRPM2: Transient receptor potential melastatin-2.

## Competing interests

The authors declare that they have no competing interests.

## Authors' contributions

LH designed the study, performed experiments, analyse the data and was involved in drafting the manuscript. MF, PB, BCdB, NK and DW performed experiments, analyzed the data and have been involved in drafting the manuscript. AT designed the study, analyzed the data and wrote the manuscript. All authors read and approved the final manuscript.
